# Gelatin Coating for the Improvement of Stability and Cell Uptake of Hydrophobic Drug-Containing Liposomes

**DOI:** 10.3390/molecules27031041

**Published:** 2022-02-03

**Authors:** Gantumur Battogtokh, Yechan Joo, Sharif Md Abuzar, Heejun Park, Sung-Joo Hwang

**Affiliations:** 1 College of Pharmacy, Yonsei University, 85 Songdogwahak-ro, Incheon 21983, Korea; gantumur.b@yonsei.ac.kr (G.B.); yechanj@naver.com (Y.J.); sumonzar@gmail.com (S.M.A.); 2 Yonsei Institute of Pharmaceutical Sciences, Yonsei University, 85 Songdogwahak-ro, Incheon 21983, Korea; 3 R&D Center, Upex-Med Co., Ltd., Anyang 14056, Korea; 4 College of Pharmacy, Duksung Women’s University, 33 Samyangro 144-gil, Seoul 01369, Korea

**Keywords:** protein coating, RGD targeting, lipid nanoparticle, paclitaxel, rhodamine B

## Abstract

Purpose: Most therapeutic agents have limitations owing to low selectivity and poor solubility, resulting in post-treatment side effects. Therefore, there is a need to improve solubility and develop new formulations to deliver therapeutic agents specifically to the target site. Gelatin is a natural protein that is composed of several amino acids. Previous studies revealed that gelatin contains arginyl-glycyl-aspartic acid (RGD) sequences that become ligands for the integrin receptors expressed on cancer cells. Thus, in this study, we aimed to increase the efficiency of drug delivery into cancer cells by coating drug-encapsulating liposomes with gelatin (gelatin-coated liposomes, GCLs). Methods: Liposomes were coated with gelatin using electrostatic interaction and covalent bonding. GCLs were compared with PEGylated liposomes in terms of their size, zeta potential, encapsulation efficiency, stability, dissolution profile, and cell uptake. Results: Small-sized and physically stable GCLs were prepared, and they showed high drug-encapsulation efficiency. An in vitro dissolution study showed sustained release depending on the degree of gelatin coating. Cell uptake studies showed that GCLs were superior to PEGylated liposomes in terms of cancer cell-targeting ability. Conclusions: GCLs can be a novel and promising carrier system for targeted anticancer agent delivery. GCLs, which exhibited various characteristics depending on the coating degree, could be utilized in various ways in future studies.

## 1. Introduction

There are several types of lipid-based nanoparticles used as drug delivery systems, such as liposomes, lipid nanoparticles, and solid lipid nanoparticles. One such example is liposomes, which are a type of nanocarrier consisting of phospholipids and cholesterol. Liposomes can encapsulate both hydrophobic and hydrophilic drugs owing to their amphipathic properties, and the surface of liposomes can be modified using functionalized lipids [1,2]. The poor physical stability of liposomes has been known to limit their wide application because it leads to the rapid aggregation and combination of membrane bilayers [3]. To enhance the stability of liposomes, polyethylene glycol (PEG), a synthetic polymer, is used to coat their surfaces. PEGylated liposomes have been known to prevent the intake of liposomes by the mononuclear phagocyte system (MPS) and greatly extend the half-life of drugs [4]. However, because PEG is not degraded by mammalian enzymes, its accumulation in normal cells can damage cellular function [5]. In addition, it has been pointed out that PEGylated liposomes have weak interactions with cancer cells because of the aqueous layers on their surfaces created by the PEG moiety [6]. Several studies have been conducted on the coating of liposomes with polymers and peptides [7,8,9]. However, few studies have reported the coating of liposomes with natural proteins, most of which have investigated albumin-coated liposomes [10,11]. Proteins are biodegradable, biocompatible, abundant, and inexpensive (reproducible by recombinant technology) materials. In addition, proteins can act as ligands targeting the receptors expressed on some cancer cells [12]. Like albumin, other proteins can be promising candidates as next-generation drug carriers.

**Figure 1:**
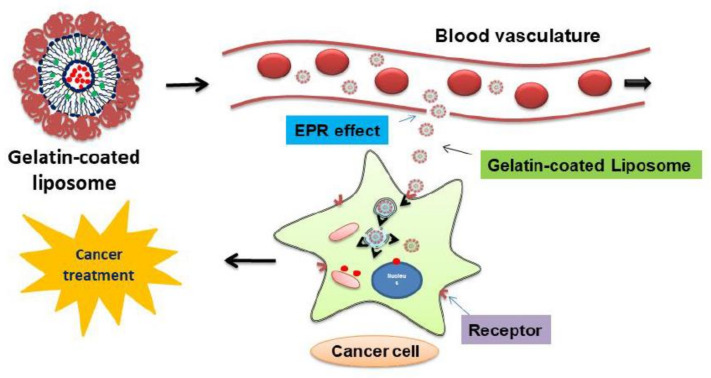
Summary of the cellular uptake of gelatin-coated liposomes.

Coating can enhance the physical stability, targeting ability, and biocompatibility of nanoparticles and reduce their toxicity. Gelatin is a natural protein that is composed of several amino acids. A recent study revealed that gelatin contains arginine, glycine, and aspartate (RGD) sequences [13,14]. These RGD sequences are ligands for the integrin receptors expressed in cancer cells [15]. Therefore, they can be applied as a coating material on nanoparticles to efficiently deliver anticancer agents into cancer cells. The mechanism of cancer cell uptake using gelatin-coated liposome formulations is divided into two stages (Figure 1). First, as the vascular endothelial cells around cancer tissues have loose structures, the nano-sized liposomes can accumulate in cancer tissues owing to the enhanced permeability and retention (EPR) effect [16]. Second, the gelatin on the surface of the liposomes could link to the integrin receptors expressed in cancer cells, thus delivering the anticancer agent directly into the cancer cells.

In this study, the hydrophobic molecule paclitaxel (PTX) as a drug or rhodamine B (RhoB) as a model payload was used. PTX is an anticancer drug used for treating various types of solid tumors and is known to inhibit tumor growth by preventing the breakdown of microtubules during cell division [17]. As PTX is a hydrophobic drug (Biopharmaceutics Classification System (BCS) class IV drug) with very low water solubility (<2 μg/mL) and poor cellular uptake, it can be encapsulated inside the phospholipid layer of liposomes and delivered to the body via an injection [18].

**Figure 2:**
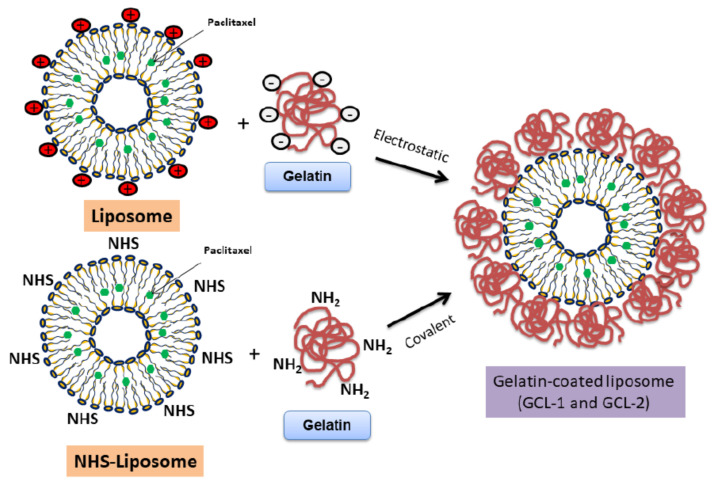
The mechanism of gelatin coating. Gelatin-coated liposome-1 was generated using charge interaction (GCL-1, **left**). Gelatin-coated liposome-2 was produced using covalent bonding (GCL-2. **right**). NHS refers to N-Hydroxysuccinimide, which readily reacts with free amine.

The purpose of this study was to prepare gelatin-coated liposomes (GCL) and further characterize their stability and cell uptake. Therefore, GCLs were prepared and compared with PEGylated liposomes in terms of size, surface charge, encapsulation efficiency (EE), physical stability, dissolution profile, and cell uptake. As shown in Figure 2, GCLs were prepared via two methods, namely electrostatic interaction and covalent linkage. In this study, we consider the advantages of GCLs over conventional liposomes and discuss their potential and limitations for commercialization.

## 2. Material and Methods

### 2.1. Materials

Hydrogenated soybean phosphatidylcholine (HSPC, Lipoid S PC-3) and N-(carbonyl-methoxypolyethylenglycol-2000)-1,2-distearoyl-sn-glycero-3-phosphoethanolamine (MPEG-2000-DSPE, Na-salt) were purchased from Lipoid GmbH (Ludwigshafen, Germany). 1,2-Distearoyl-sn-glycero-3-phosphoethanolamine-N-hydroxysuccinimide (DSPE-NHS) was bought from Nano Soft Polymers Co., Ltd., (Winston-Salem, NC, USA). Cholesterol, stearylamine, and gelatin from bovine skin (Gel, Type B) were purchased from Sigma-Aldrich (St. Louis, MO, USA). PTX was purchased from Tokyo Chemical Industry Co., Ltd. (Tokyo, Japan). RhoB was obtained from Sigma-Aldrich (St. Louis, MO, USA). The Float-A-Lyzer G2 (molecular weight cut-off (MWCO): 100 kDa) was purchased from Spectrum Laboratories, Inc. The Pur-A-Lyzer Mini (MWCO: 12 kDa) was purchased from Sigma-Aldrich (St. Louis, MO, USA). The Bradford protein assay kit (Pierce Coomassie Protein Assay Kit) was purchased from Thermo Scientific (Waltham, MA, USA). Milli-Q quality purified water (Milli-Q Reference; Millipore, Molsheim, France) was used throughout the study. All organic solvents were purchased from Samchun Chemicals (Gyeonggi-do, Korea). All other chemicals were of reagent grade. The NanoDeBee homogenizer (BEE International Co., Ltd.; South Easton, MA, USA) was used for the preparation of liposomes.

### 2.2. Preparation of PTX-Encapsulating Plain Liposomes

Liposomes were prepared by referring to several existing studies on thin-film hydration methods [19,20]. Briefly, lipids, cholesterol, and PTX were dissolved in 9 mL of chloroform in a 100 mL round-bottomed flask at 40 °C. The quantities of each formulation material are summarized in Table 1. After complete dissolution, the organic solvent was removed using a rotary evaporator at 40 °C for 2 h. The thin film was hydrated with 10 mL of a 50 mM potassium phosphate buffer (pH 7.0) at 70 °C in a water bath with rotation for 30 min. The hydrated mixture was then extruded using a NanoDeBee homogenizer (BEE International Co., Ltd.; South Easton, MA, USA) at 1500 bar for 10 cycles to produce small unilamellar vesicles (SUV). To remove non-encapsulated PTX, the liposome suspension was centrifuged at 1000 rpm for 10 min at 25 °C. Size and zeta potential were analyzed using a zeta potential and particle size analyzer instrument after a five-fold dilution.

### 2.3. Encapsulation Efficiency

The PTX content in liposomes was determined using high-performance liquid chromatography (HPLC) based on the standard curve described in published articles [21,22,23]. To measure their EE and encapsulation capacity, the liposomes were disrupted with acetonitrile, and the drug content was determined using a reverse-phase HPLC system (Agilent Technologies; Palo Alto, CA, USA) equipped with a 4.6 × 250 mm, 5 μm Sepax BR-C18 column (Sepax Technologies, Inc.; Newark, DE, USA) at 30 °C and a flow rate of 1 mL/min, using a mixture of acetonitrile and water 60:40 (*v*/*v*) as the mobile phase. An injection volume of 20 μL of PTX was used, and it was detected at a wavelength of 227 nm. The concentration of PTX was calculated from a calibration curve constructed using solutions containing varying concentrations of PTX (0.01–100 μg/mL).

The EE of each formulation was calculated using the following equation:

(1) $$\mathrm{EE}\;\left(\%\right)=\frac{\mathrm{Feed}\;\mathrm{PTX}-\mathrm{found}\;\mathrm{PTX}\;}{\mathrm{Feed}\;\mathrm{PTX}}\times 100$$

### 2.4. Fabrication of Gelatin-Coated Liposomes (GCL)

The prepared plain liposomes were coated with gelatin in phosphate-buffered saline (PBS) (pH 8.0) via electrostatic interaction (GCL-1) or covalent bonding (GCL-2). For the GCL-1, the liposome solution was mixed with various concentrations of a gelatin solution (500, 1000, 2000, and 10,000 µg/mL) in a 1:1 (*v*/*v*) ratio of liposome solution to gelatin solution and incubated at 25 °C and 50 rpm for 18 h. For GCL-2, the N-hydroxysuccinimide (NHS) liposome solution (pH 7.0) was mixed with two different amounts of the gelatin solution (pH 8) and incubated at 25 °C and 50 rpm for 24 h. After that, to remove free gelatin, the solution was dialyzed in a membrane tube (MWCO 100 kDa) in PBS (pH 7.4) for 4 h. The gelatin-coating efficiency was measured using a Bradford protein assay [24]. Briefly, 10 μL of the sample was added to 300 μL of reagent and gently shaken. After 10 min, the absorbance was measured at 595 nm using a UV spectrophotometer. The size and zeta potential of the liposomes were measured using a zeta-potential and particle size analyzer (ELSZ-1000; Otsuka Electronics Co; Osaka, Japan). Scattered light was detected at 23 °C and an angle of 90°. A viscosity value of 0.933 mPa and a refractive index of 1.333 were used for data analysis.

### 2.5. Differential Scanning Calorimetry (DSC)

A DSC system (AUTO-DSC Q2000; Ta Instruments; New Castle, DE, USA) was used to characterize the phase transition behavior of the materials and liposomes. Briefly, 3–5 mg of lyophilized liposomes were placed in standard aluminum pans. The measurements were performed in the temperature range of 0 to 200 °C, with a heating and cooling rate of 10 °C/min. A blank pan was used as a reference. The TA Universal analysis software was utilized to identify the phase transition temperature (T_m_) and melting point.

### 2.6. Fourier-Transform Infrared Spectroscopy (FT-IR)

The GCLs were freeze-dried, and the powder samples were analyzed using FT-IR (Cary 630; Agilent Technologies; Santa Clara, CA, USA) at 4 cm^−1^ resolution. The FT-IR spectra of dispersed samples were measured in the 600–4000 cm^−1^ region.

### 2.7. Physical Stability Analysis

The stability of the GCLs was determined via size measurements using Dynamic Light Scattering (DLS). To analyze the time-dependent physical stability, the sizes of the PEGylated liposomes, GCL-1, and GCL-2 were measured at 4 °C and 25 °C for 8 weeks in PBS with 0.5% Tween 80. Before each measurement, the samples were shaken by inverting the tube. The stability of the GCLs was compared with that of the PEGylated liposomes.

### 2.8. In Vitro Release Study

The in vitro release profile was investigated using previously reported methods with minor modifications [19,23,25]. A solution of PEGylated liposomes and GCLs (200 μL, 250 μg/mL of PTX) was prepared and added to a Pur-A-Lyzer Mini tube with an MWCO of 12 kDa (Sigma-Aldrich; St. Louis, MO, USA). The tubes were immersed in 50 mL of PBS (pH 7.4) containing 0.1% (*w*/*v*) Tween 80 and incubated at 37 °C with the rotation at 50 rpm. The dissolution medium (1 mL) was collected at various time points (1, 2, 3, 4, 6, 9, 12, and 24 h) and replaced with 1 mL of a fresh medium at 37 °C. The amount of released PTX was determined using HPLC.

### 2.9. Cell Culture and Cellular Uptake Study

HeLa cells were cultured in Dulbecco’s modified Eagle’s medium (DMEM) supplemented with 10% fetal bovine serum (FBS) (2 g/L for RPMI-1640 and 4.5 g/L for DMEM) and 1% penicillin/streptomycin in humidified air with 5% CO_2_ at 37 °C. In the cell-uptake study, to analyze liposome uptake by the cells, RhoB (a fluorescence agent) was used instead of PTX because of their similar properties. To assess the cellular uptake of free RhoB or RhoB-loaded liposomes, RhoB was added to the formulation at a 1% molar ratio of the lipid. The cells were seeded in 96-well plates at a density of 2 × 10^4^ cells/well in the culture medium (100 μL) and incubated for 24 h. After 4 h of treatment with 10 mg/mL of free RhoB, RhoB-loaded PEGylated liposomes, or RhoB-loaded GCLs, the cells were rinsed twice with Dulbecco’s phosphate-buffered saline (DPBS) and detached. To determine the cellular uptake of RhoB-GCLs, the fluorescence signal of the RhoB was measured using a microplate reader (Infinite^®^ 200 PRO; Tecan, Grödig, Austria) linked to a fluorescence detector (578 nm).

### 2.10. Statistical Analysis

All of the studies were carried out in triplicate, and the results were expressed as mean ± standard deviation (S.D.). The statistical significance of the data was analyzed using the Student’s t-test. In all cases, *p* < 0.05 was considered to be statistically significant.

## 3. Results and Discussion

### 3.1. Preparation of PTX-Encapsulating Plain Liposomes

Three liposome formulations (PEGylated liposomes, GCL-1, and GCL-2) were prepared using the film-forming and hydration methods. GCL-1 showed a positive charge due to stearylamine, whereas GCL-2 showed a negative charge. Hydration was performed at 70 °C above the glass transition temperature (T_g_) of the phospholipids. After sufficient hydration, large-sized multilamellar vesicles (MLVs) were formed in the liposome dispersion. For intravenous injection (IV), the size of the liposomes should be small and uniform. Thus, the liposome dispersion was extruded 10 times through the NanoDeBee to reduce the size of the liposomes. The EE of liposomes is an important indicator for clinical applications. Usually, liposomes with EEs of more than 80% of the drug are considered effective [26]. The PEGylated liposomes showed 82.5% drug EE. GCL-1 and GCL-2 showed 92.0% and 92.7% of drug EE, respectively, both indicating well-encapsulated liposomes. Significantly lower EE percentages, which are not listed in Table 2, were observed in formulations prepared with DSPE at a 10% molar ratio. It is assumed that this occurs because DSPE tends to self-aggregate in the hydration stage, as the hydration repulsion between its particles is too small. Owing to the aforementioned reasons, the thin film did not form well, and the EE of the PTX was found to be approximately 18.6%. One of the most important factors determining EE is the amount of hydrophobic material. Cholesterol is added to increase the rigidity of the liposome structure [27]. However, EE can be reduced when the amount of cholesterol or the drug is too high, as cholesterol and the drug compete for the hydrophobic region of the lipid bilayer [28]. In this study, the cholesterol concentration was set at 40% to achieve high stability without significantly lowering the EE of the drug. The EE of RhoB in the liposomes was calculated to be more than 90%.

### 3.2. Fabrication of Gelatin-Coated Liposome (GCL)

Considering the high stability of gelatin under weakly basic conditions, we used a phosphate buffer (pH 8.0) as a solvent [29]. For GCL-1 and GCL-2, the coating was induced by different mechanisms. In the case of GCL-1, liposomes were coated with gelatin using electrostatic interaction. Stearylamine was added to impart a positive charge to the liposomes so that the negatively charged gelatin at physiological pH could coat the surface of the liposomes. In the case of GCL-2, gelatin coating was achieved via covalent bonding. The phospholipid used in our study has an NHS group attached to the hydrophilic head of DSPE. Gelatin contains several amines; therefore, when the amino group interacts with the NHS group of the liposome between pH 7 and 9, they form a strong amide bond, as shown in Figure S1 (Supplementary Materials) [30]. Unreacted gelatin molecules were removed using a Float-A-Lyzer G2. As gelatin has a molecular weight of 60 kDa, a semi-permeable membrane with an MWCO of 100 kDa was used. The coating efficiency of gelatin on liposomes was confirmed using the Bradford protein assay.

### 3.3. Characterization of GCL-1 and GCL-2

**Figure 3:**
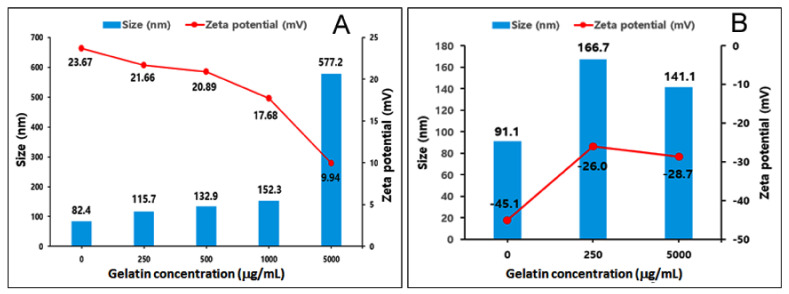
Size and zeta potential of GCL-1 (**A**) and GCL-2 (**B**) according to gelatin concentration.

The size, polydispersity index, and zeta potential of the liposome formulations are shown in Table 2. The gelatin concentration of both formulations (GCL-1 and GCL-2) was 250 μg/mL. In the case of GCL-1, when the thickness of the gelatin on the surface of the liposomes increased, the zeta potential decreased (Figure 3A). On the contrary, in GCL-2, an increase in the concentration of gelatin did not significantly affect the size and zeta potential of the liposomes (Figure 3B). This is presumably owing to its limited number of covalent bonds, unlike GCL-1, which was coated using electrostatic interaction. Nanoparticles with a diameter of less than 200 nm are known to transfer drugs to cancer tissues with a leaky structure of vascular endothelial tissue via the EPR effect [31]. Therefore, small-sized liposomes, such as PEGylated liposomes and GCL-2, are advantageous because of their accumulation in cancer tissues when injected into the bloodstream. As GCL-1 was relatively large in size owing to coating with a high concentration of gelatin, sufficient consideration should be given during intravenous injection.

Particles with zeta potentials above30 mV or under -30 mV have an electrical repulsive force, thus decreasing their tendency to aggregate and increasing their stability in the colloidal state [32]. PEGylated liposomes show near-zero zeta potentials; thus, their colloidal stability is expected to be relatively low [33]. The long-term stability of GCL-1 and GCL-2 in suspension is expected to be superior to that of PEGylated liposomes.

Figure S2 (Supplementary Materials) shows the T_g_ and melting point of each substance, as measured using DSC. Melting peaks were observed at 147.96 °C for cholesterol and 85.66 °C for HSPC. The peaks of T_g_ for DSPE-NHS and DSPC-PEG2000 appeared at 56.86 °C and 53.88 °C, respectively, but the T_g_ of HSPC was not obtained owing to low calories.

Measurement of the T_g_ of liposome components using DSC is critical. T_g_ is the temperature at which the transition occurs from the ordered gel phase to the lipid’s physical state. As the temperature in the hydration step must be higher than the T_g_ of the lipids, the experiment was performed at 70 °C.

**Figure 4:**
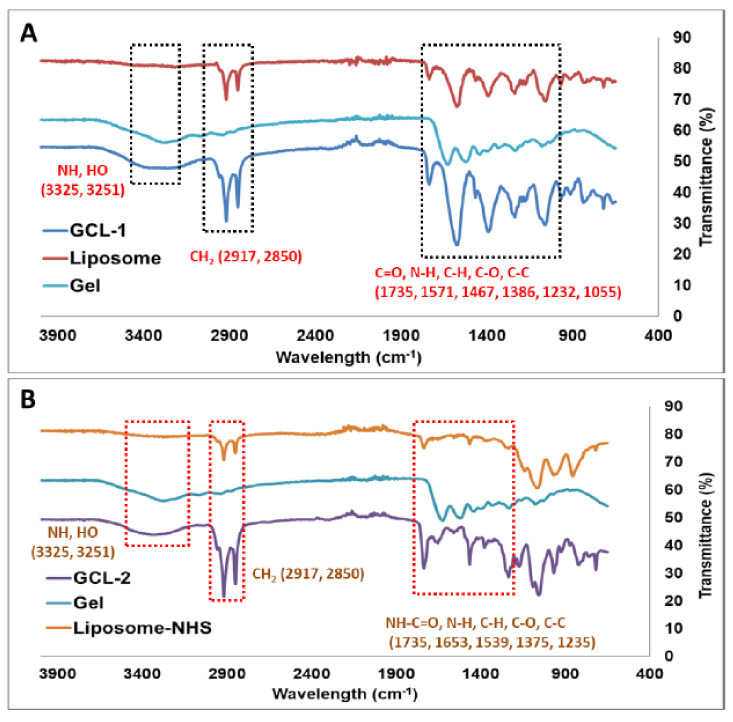
Graph of Fourier-transform infrared spectroscopy data comparing gelatin-coated liposome-1 (GCL-1 (**A**) and CGL-1 (**B**)) before coating (**up**) and after coating (**down**).

Figure 4 shows the differences in the FT-IR signal before and after the gelatin coating of GCL-1 and GCL-2. As shown in Figure 4A,B in the graph, before coating, a peak corresponding to the CH_2_–CH_2_ bond was observed at 2850 and 2917 cm^−1^, and a peak corresponding to the C=O bond was observed at 1735 cm^−1^. Additional peaks corresponding to C–H, C–O, and N–H were observed at 1571, 1386, 1232, and 1055 cm^−1^, respectively. As gelatin was coated on the surface of the liposomes (GCL-1 and GCL-2), a new wide peak at around 3300 cm^−1^ appeared that corresponded to hydroxyl and amide bonds (HO, NH) in the gelatin moiety, confirming that the gelatin coating was on the liposomes. In addition, peaks for C=O, N-H, C-H, C-O, and C-C were observed at 1735, 1571, 1467, 1386, 1232, and 1055 cm^−1^, respectively. In the case of GCL-2 (Figure 4B), which was obtained via covalent bonding between NHS-bound lipids and the amine groups of gelatin, an additional peak at 1653 cm^−1^ corresponding to the carbonyl group (C=O) of the amide bond was observed, indicating the successful coating of gelatin on the liposome.

**Figure 5:**
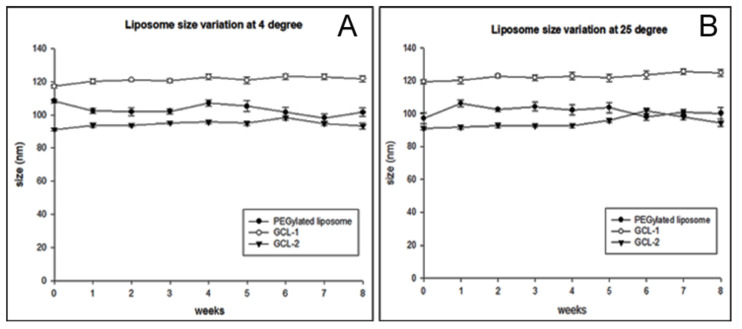
(**A**) Particle size of liposomes during storage at 4 °C for 8 weeks; (**B**) Particle size of liposomes during storage at 25 °C for 8 weeks.

To evaluate the stability of the liposomes, the particle size of each formulation was measured for 8 weeks using DLS at 4 °C and 25 °C (Figure 5). The PEGylated liposomes, GCL-1, and GCL-2 all showed no big difference in size at both 4 °C and 25 °C for 8 weeks, and the polydispersity index remained similar. These results confirmed that the gelatin coating on liposomes retained liposome stability for a long time.

### 3.4. In Vitro Release Study

Prior to the release study, to determine the amount of soluble PTX in the release medium, the solubility of PTX in various solvent concentrations (Tween 80 in PBS) was measured to determine sink conditions. The results are shown in Figure S3 (Supplementary Materials). The solubility of PTX in PBS (1×) was found to be 1.73 μg/mL, and it increased with the addition of Tween 80. When 0.5% Tween 80 (*w*/*v*) was added to PBS, PTX solubility increased to 17.19 μg/mL. However, 0.1% of Tween 80 (*w*/*v*) in PBS was sufficient to create a sink condition, which is a dissolution media volume that has the ability to dissolve at least 3-5 times the amount of payload than the saturation volume. Because the molecular weight of PTX is 853.906 Da, dialysis tubes with an MWCO of 12 kDa were used.

**Figure 6:**
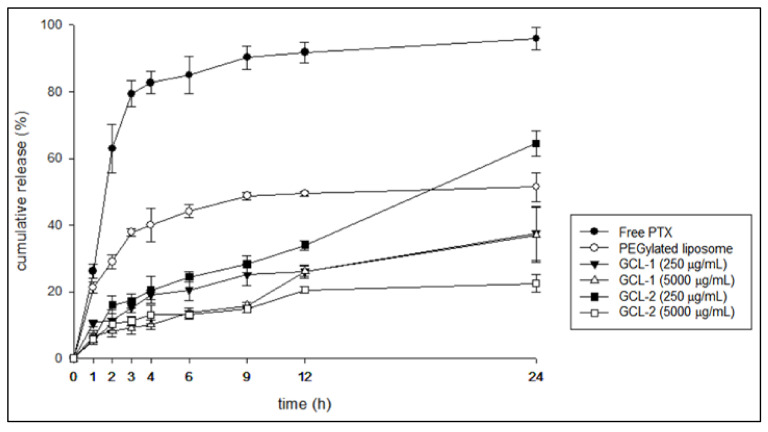
In vitro release profiles of the liposome formulations and free PTX.

Figure 6 shows the 24 h in vitro release profiles of free PTX, PTX-PEGylated liposomes, GCL-1, and GCL-2 in physiological conditions. More than 80% of the free PTX was released at 4 h. The GCLs were found to inhibit the initial release of PTX compared to the PEGylated liposomes. It appeared that the thick gelatin on the surface of the liposomes delayed the initial drug release rate. Drug dissolution also differed according to the gelatin concentration and coating mechanism. The covalently coated GCL-2 exhibited a slower release than the physically coated GCL-1 (generated via electrostatic interaction), and this effect increased with increasing gelatin concentrations. In view of the slower release of PTX from GCL-2 despite the larger size of GCL-1, the dissolution behavior of GCLs cannot simply be explained by the thickness of the coating layer. Gelatin is one of the most widely used protective colloids [34]. Gelatin coating on the surface of liposomes significantly increased their physical stability. This effect was assumed to be stronger when the coating was achieved via strong interactions, such as covalent bonds, rather than weak electrostatic interactions.

### 3.5. Cell Uptake Study

**Figure 7:**
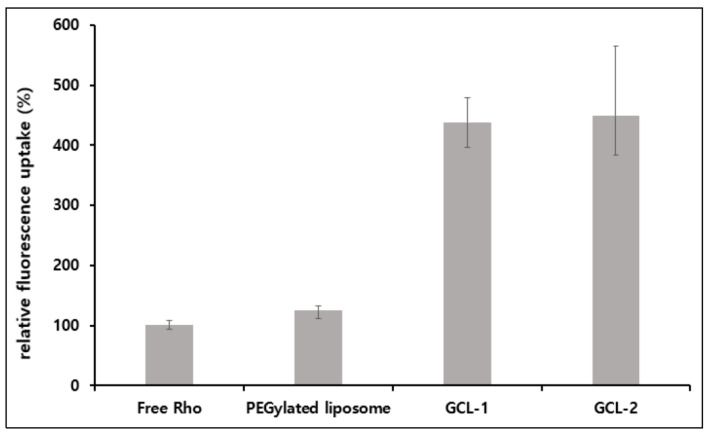
Relative fluorescence uptake among free rhodamine B and liposome formulations.

As shown in Figure 7, a cell uptake study was conducted to confirm the effective delivery of drugs into cancer cells (HeLa cells) [35,36]. For the cell uptake study using liposomes, we replaced PTX with RhoB, which is a hydrophobic fluorophore with a structure similar to that of PTX, because the fluorescence emission agent was needed for analysis using confocal microscopy. The cultured cells were treated with free RhoB and RhoB-loaded liposome formulations. Next, the fluorescence in the HeLa cells was analyzed after 4 h. The relative fluorescence uptake was evaluated based on the fluorescence in the free RhoB-treated cells. PEGylated liposome-treated cells showed 124.9% fluorescence compared to the free RhoB-treated cells and did not show significant differences from free RhoB-treated cells. On the contrary, cells treated with GCL-1 and GCL-2 showed 437.1% and 447.7% fluorescence, respectively, compared to the cells treated with free RhoB. The level of cellular fluorescence was associated with the fact that the liposomes were absorbed into the cell, thus indicating efficient drug delivery. The low cell-uptake efficiency of the PEGylated liposomes was due to their reduced interaction with cells, which is caused by the water layer of PEG formed on the surface of the liposomes. In contrast, GCL-1 and GCL-2 showed 3.50- and 3.58-times higher uptake efficiencies than the PEGylated liposomes, respectively. It is considered that the RGD sequences in the gelatin on the surface of GCLs can affect the uptake efficiency because they bind to the integrin receptors expressed on HeLa cells [14,15,35].

## 4. Conclusions

To enhance the targeting ability of liposomal carrier systems, we developed GCLs in this study. The gelatin coating on the liposomes worked as intended, and the GCLs exhibited different properties depending on the coating mechanism, namely electrostatic interaction or covalent bond formation. The small-sized formulations of GCLs (both liposomes coated using covalent and electrostatic interactions) were stable for 8 weeks at 4 °C and 25 °C. Gelatin was originally used to increase the affinity of the liposomes to cancer cells, but it also conferred on the liposomes a sustained drug-release property. Gelatin appeared to increase the physical stability of the liposomes by acting as a protective colloid. The ability to control the drug-release pattern according to the degree of coating offers many possibilities. Cell-uptake studies showed the superior cancer cell-targeting ability of GCLs over PEGylated liposomes. This aspect of GCLs could allow for the selective delivery of a payload drug into cancer cells after intravenous injection into the bloodstream. The mechanism for efficient cell uptake of GCLs could be explained on the basis of receptor-mediated endocytosis through the RGD sequences and integrin receptors in HeLa cells. These results should be confirmed in more detail using in vivo studies. Although coating liposomes with natural proteins (like gelatin) is an alternative candidate to PEGylation, there are some limitations to direct commercialization due to their immunogenicity, manufacturing complexity, and cost. Therefore, further studies should focus on overcoming these limitations.

## Figures and Tables

**Table 1:** Formulations of liposomes. Lipid 1 refers to HSPC, and lipid 2 refers to DSPE-PEG2k or Stearylamine or DSPE-NHS.

	PEGylated Liposomes	GCL-1	GCL-2
Lipid 1	HSPC 50 mg	HSPC 50 mg	HSPC 50 mg
Lipid 2	DSPE-PEG2k 14 mg	Stearylamine 3.23 mg	DSPE-NHS 10.4 mg
Cholesterol	17.2 mg	15.8 mg	15.8 mg
PTX	4 mg	4 mg	4 mg
Molar ratio (Lipid 1:Lipid 2:Chol)	57:4:39	55:10:35	55:10:35

Abbreviations: GCLs, Gelatin-coated liposomes; PTX, paclitaxel; Chol, cholesterol.

**Table 2:** Main characteristics of the liposomes. The gelatin concentration of GCL-1 and GCL-2 was 250 μg/mL.

	PEGylated Liposomes	GCL-1	GCL-2
Size (nm)	113.3 ± 1.7	115.7 ± 5.2	105.0 ± 3.1
Polydispersity index	0.241	0.250	0.250
Zeta potential (mV)	0.72 ± 0.14	21.7 ± 2.9	−28.1 ± 0.5
Encapsulation efficiency (%)	82.5 ± 2.5	92.0 ± 5	92.7 ± 5.5

## Supplementary Materials

The following are available online, Figure S1: N-Hydroxysuccinimide (NHS) and amine reaction which forms amide bond between gelatin and carboxyl moiety on liposome, Figure S2: DSC diagram of phospholipids and cholesterol, Figure S3: Solubility of paclitaxel with various concentrations of tween 80.

## Sample Availability

Samples of the compounds are not available from the authors upon request.
