# Supplementary material for: Gelatin Coating for the Improvement of Stability and Cell Uptake of Hydrophobic Drug-Containing Liposomes
Source: Molecules. 2022 Feb 3;27(3):1041. doi: 10.3390/molecules27031041 (PMC8838450; doi:10.3390/molecules27031041)
Supplement: Supplementary file 1 [file molecules-27-01041-s001.zip › molecules-1547817-supplementary.pdf]

molecules

MDPI

Supplementary Materials

Gelatin Coating for the Improvement of Stability and Cell Uptake of Hydrophobic Drug-Containing Liposomes

Gantumur Battogtokh [1,2,3,†], Yechan Joo [1,2,†], Sharif Md Abuzar [1,2], Heejun Park [4,*] and Sung-Joo Hwang [1,2,*]

[1] College of Pharmacy, Yonsei University, 85 Songdogwahak-ro, Yeonsu-gu, Incheon 21983, Korea; gantumur.b@yonsei.ac.kr (G.B.); yechanj@naver.com (Y.J.); sumonzar@gmail.com (S.M.A.);
[2] Yonsei Institute of Pharmaceutical Sciences, Yonsei University, 85 Songdogwahak-ro, Yeonsu-gu, Incheon 21983, Korea
[3] R&D Center, Upex-Med Co., Ltd, 14056, Anyang, Korea
[4] College of Pharmacy, Duksung Women's University, 132-714 Seoul, Korea
* Correspondence: heejunpark@duksung.ac.kr (H.P.); sjh11@yonsei.ac.kr (S.-J.H.);
† These authors contributed equally to this work.

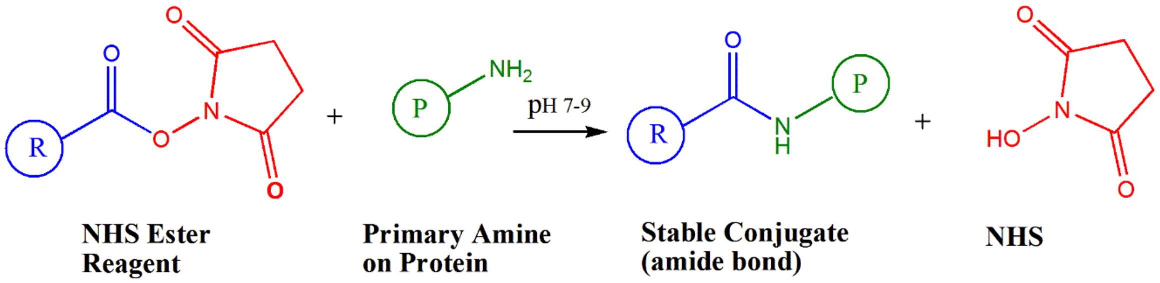

Figure S1. N-Hydroxysuccinimide (NHS) and amine reaction that forms amide bond between gelatin and carboxyl moiety on the liposome.

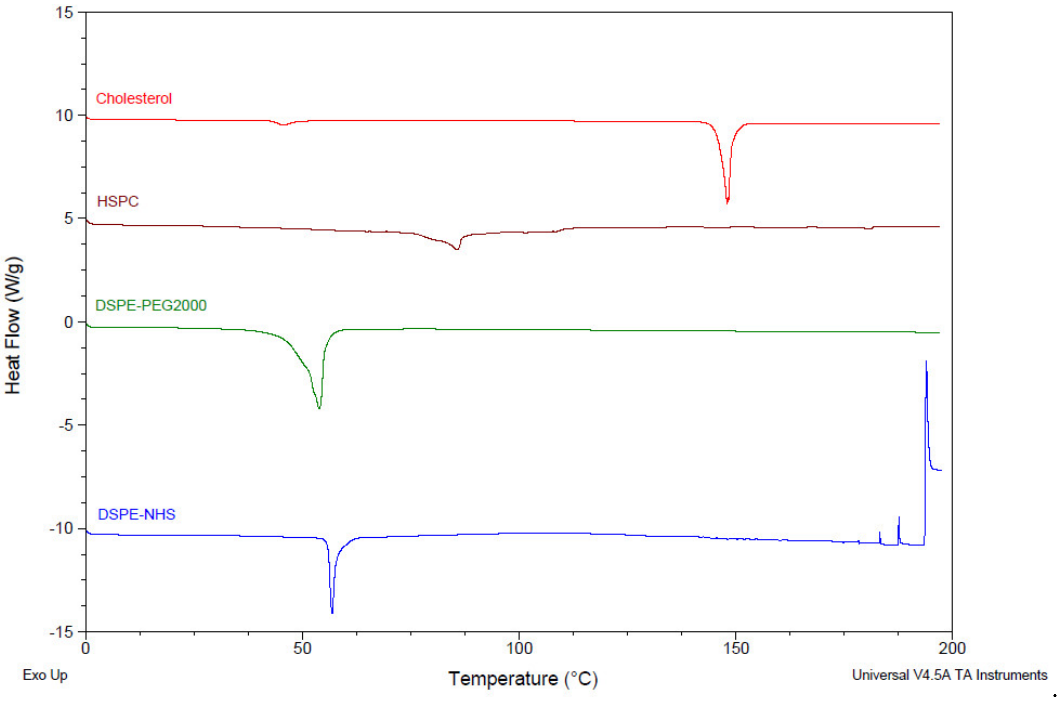

Figure S2. DSC diagram of phospholipids and cholesterol.

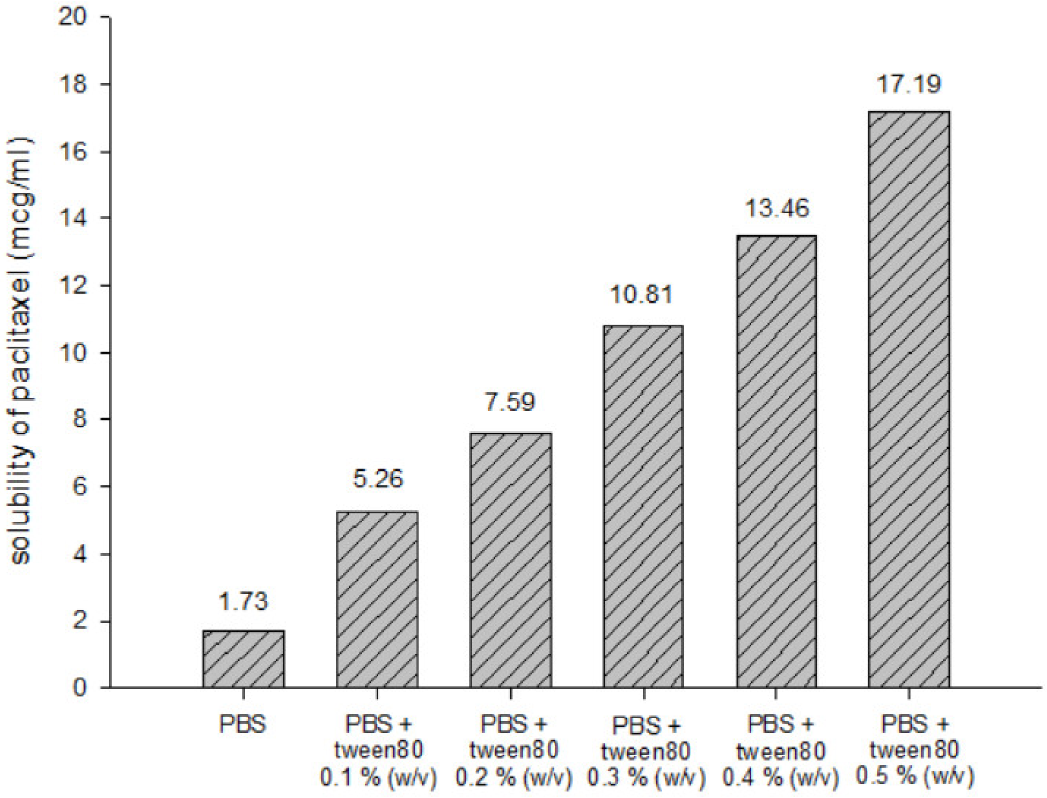

Figure S3. Solubility of paclitaxel with various concentrations of tween 80.
